# Neutrophil-to-Lymphocyte Ratio and Treatment Failure in Peritoneal Dialysis-Associated Peritonitis

**DOI:** 10.3389/fmed.2021.699502

**Published:** 2021-07-26

**Authors:** Peng He, Li-jie He, Chen Huang, Jin-ping Hu, Shi-ren Sun

**Affiliations:** Department of Nephrology, Xijing Hospital, The Fourth Military Medical University, Xi'an, China

**Keywords:** neutrophil-to-lymphocyte ratio, peritoneal dialysis-associated peritonitis, peritoneal dialysis, treatment failure, catheter removal

## Abstract

**Objective:** We sought to explore if there is an association between neutrophil-to-lymphocyte ratio (NLR) and treatment failure in patients with peritoneal dialysis-associated peritonitis (PDAP).

**Methods:** Our cohort involved 337 episodes of PDAP experienced by 202 patients who were undergoing continuous ambulatory peritoneal dialysis at a single center from 1 July 2013 to 30 June 2018. The exposures were log-transformed NLR and a categorical variable grouped by the tertiles of NLR levels (T1, <3.75; T2, 3.75–6.53; and T3, >6.53) at baseline. Generalized estimating equation (GEE) and restricted cubic spline (RCS) analyses were done to determine the association between NLR and treatment failure, defined as catheter removal or all-cause mortality during therapy.

**Results:** After adjusting for other potential predictors, the log-transformed NLR exhibited an incremental relationship with the risk of treatment failure (odds ratio, 1.82; 95% confidence interval, 1.05–3.15). RCS analyses showed that the relationship was positively and linearly correlated (*P* for nonlinearity = 0.104). As a three-level categorical variable, in reference to T1, the T3 of NLR showed a 3.41-fold increased venture of treatment failure in fully adjusted model. Subgroup analyses suggested that the prognostic relevance of NLR in PDAP was particularly significant in gram-negative peritonitis.

**Conclusions:** A greater level of NLR at baseline was remarkably associated with a higher incidence of treatment failure among PDAP episodes regardless of other potential risk factors.

## Introduction

Peritoneal dialysis-associated peritonitis (PDAP), as one of the most common and severe complications, remains a crucial reason for technical failure among PD patients, responsible for about 22% catheter removal, 18% transfer to hemodialysis (HD), and 2–6% mortality (1, 2). Specifically, persistent peritonitis, an inadequate response to treatment, and the inflammatory state inherent in PD patients may result in extension of hospitalization time, increase of hospitalization expense, and impairment of peritoneal structure and function (3–6). The International Society for Peritoneal Dialysis (ISPD) recommends that the PD catheter should be removed promptly in refractory/relapsing/recurrent/repeat peritonitis episodes, defined as failure of the PD effluent to clear up after 5 days of appropriate antibiotics, and only a small percentage of patients can restart PD therapy (7). Despite the guidelines for PDAP, there are still quite puzzling differences in the treatment outcomes of peritonitis in many centers and countries. In view of the poor outcomes, early warning and decision-making are needed in clinical practice. Furthermore, existing studies have highlighted the forecasting value of novel biomarkers for adverse outcomes of peritonitis (8–10).

Neutrophil-to-lymphocyte ratio (NLR) is obtained simply by dividing the absolute neutrophil count by the absolute lymphocyte count in peripheral blood. Recently, NLR has been reported to be associated with inflammation in end-stage renal disease (ESRD) including both HD and PD patients, and to estimate survival in these patients (11–15). However, to date, there has been little evidence to show a relationship between NLR and adverse outcomes in patients with PDAP, and the prognostic impact of NLR in this population remains unclear. Therefore, in the current study, we sought to investigate the association between increased NLR and treatment failure in patients with PDAP.

## Methods

### Episodes

This single-center, retrospective observational study was conducted at the PD center of Xijing Hospital, Xi'an, China. Data regarding all episodes of PDAP from 1 July 2013 to 30 June 2018 were collected by reviewing case records. All patients received continuous ambulatory peritoneal dialysis (CAPD) using lactate-buffered glucose dialysis solution through Tenckhoff PD catheters with a twin-bag connection system. Our research was done in accordance with the principles of the Declaration of Helsinki. Relevant information was processed anonymously, and personal identifiers were completely wiped off. The study was approved by the Ethics Committee of Xijing Hospital (no. KY20163154-1).

Peritonitis was diagnosed independently by two physicians according to the 2016 ISPD guidelines if at least two of the following items were met: (1) abdominal pain and/or cloudy dialysate effluent; (2) dialysate white blood cell counts (WBCs) >100/μl (after a dwell time of at least 2 h), with polymorphonuclear of >50%; and (3) positive dialysate effluent culture (7). The exclusion criteria included episodes of (1) fungal, (2) polymicrobial, and (3) mycobacterial peritonitis and (4) episodes without bacterial cultures or missing data.

### Clinical Data, Definitions, and Outcomes

In our PD center, when PDAP was suspected, 10 ml dialysate effluent (intraperitoneal retention time of at least 4 h) was collected under strict aseptic operation for routine examination. Five to 10 ml dialysate effluent was injected into two (aerobic and anaerobic) blood culture bottles for microbiology and antibiotic susceptibility tests. After three consecutive samples were collected, empirical antimicrobial treatment was initiated, including first-generation cephalosporin or vancomycin combined with third-generation cephalosporin or aminoglycoside. Meanwhile, information regarding signs, symptoms, and antibiotic dose were recorded daily. The medication regimen was modified according to the culture results and antibiotic susceptibility. Catheter removal was performed in patients with refractory peritonitis (failure of the dialysate effluent to clear up after 5 days of appropriate antibiotic treatment), refractory exit-site or severe tunnel infection, or deterioration of the clinical condition as judged by the physician.

Routine blood test was done using an automatic hematology analyzer (XN-9000, Sysmex, Kobe, Japan) prior to antibiotic therapy. NLR was calculated as the ratio of neutrophils to lymphocytes. Other clinical characteristics included age, gender, PD duration, comorbidities, residual urine volume, bacterial culture result, red blood cell counts (RBCs), WBCs, platelet, hemoglobin, serum albumin, serum creatinine, serum uric acid, serum cholesterol, serum potassium, serum phosphorus, serum ferritin, and dialysate WBCs on day 3 after initiating antibiotic therapy. The baseline laboratory data were obtained within 1 week prior to antibiotic treatment.

The primary endpoint was treatment failure, defined as catheter removal (including a temporary or permanent switch to HD) or all-cause mortality.

### Statistical Analyses

Normally distributed data were expressed as mean ± standard deviation (SD), while skewed data were expressed as median with interquartile range (IQR). Categorical variables were presented as numbers (*n*) with percentage (%). Comparisons of NLR values between various outcomes were done by Kolmogorov–Smirnov tests. The eligible episodes were categorized by tertiles (T1, T2, and T3) of NLR. As a continuous variable, NLR was log-transformed due to the positively skewed distribution. Generalized estimation equation (GEE) analyses, which accounted for multiple peritonitis episodes in the same patient, were done to evaluate the covariate-adjusted relationship between NLR levels and the risk of treatment failure events. The results were reported as odds ratios (ORs) with 95% confidence intervals (95% CIs). The variables with a bilateral *P* value of <0.25 in univariable models were regarded as potential predictors of treatment failure and adjusted in multivariable models. For determination of the association of NLR levels with the adverse outcome, restricted cubic spline (RCS) was used to model the level of NLR after multivariable adjustment.

Sensitivity analysis was performed on the basis of the endpoint of catheter removal. Preplanned subgroup analyses were done by age (>60 vs. ≤60 years), gender, PD duration (>12 vs. ≤12 months), serum albumin (>25 vs. ≤25 g/l), and infection type. *P* < 0.05 (two-sided) were considered statistically significant. All data were analyzed using the statistical software Stata Edition 15.0 (StataCorp, College Station, TX, USA).

## Results

### Episodes and Outcomes

During the period of our study, the initial cohort involved 365 episodes of PDAP. Twenty-eight episodes were excluded due to the following reasons: 12 episodes were polymicrobial peritonitis, 10 were fungal peritonitis, and 6 were with missing data. In the end, 337 episodes experienced by 202 patients were eligible for statistical analyses. The median (IQR) age was 45 (21) years, and 198 (58.5%) were male. The median (IQR) duration of PD was 16 (25) months. Forty-six (13.6%) episodes, including 39 (11.6%) catheter removal and 7 (2.1%) deaths, were identified with treatment failure.

**Figure 1:**
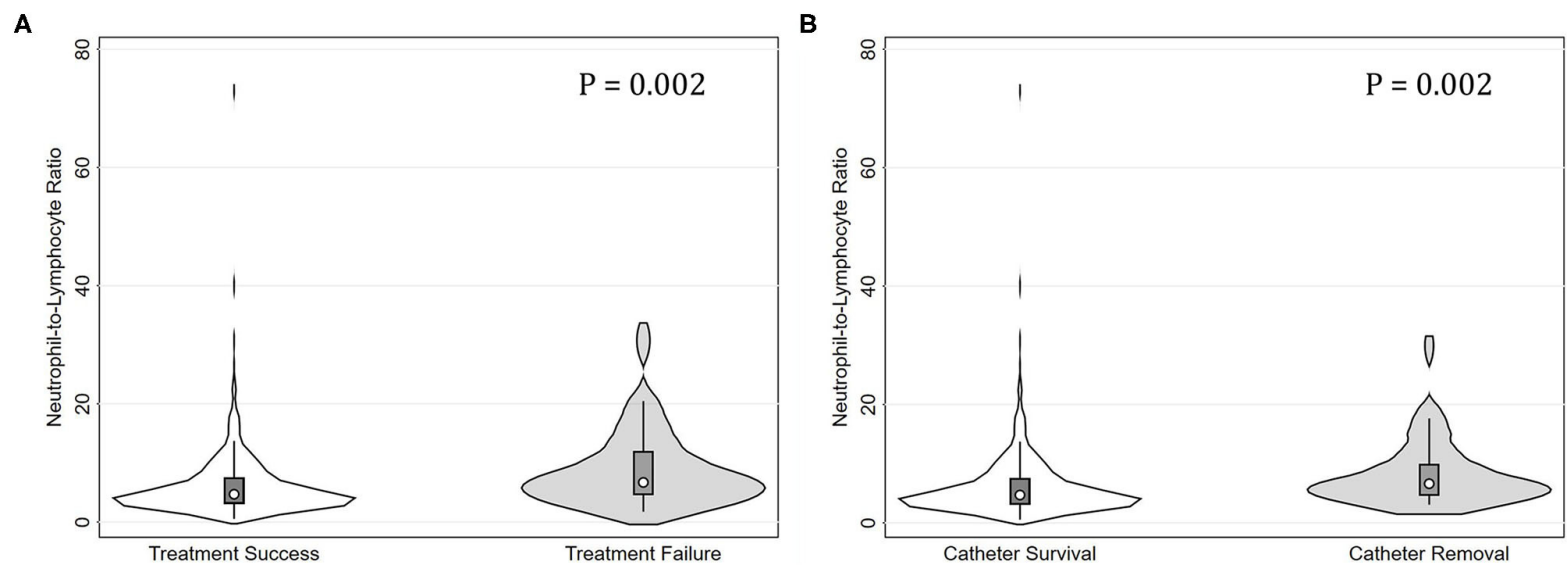
Violin plots for neutrophil-to-lymphocyte ratio (NLR) levels in peritonitis episodes grouped by treatment failure and catheter removal. **(A)** Episodes with treatment failure had significantly higher NLR levels than those with treatment success (*P* = 0.002). **(B)** Episodes with catheter removal had significantly higher NLR levels than those with catheter survival (*P* = 0.002).

The median (IQR) NLR was 4.86 (4.61). Table 1 summarizes the clinical characteristics of all the eligible episodes grouped by the tertiles of NLR (T1, <3.75; T2, 3.75-6.53; and T3, >6.53). Among them, 113, 112, and 112 episodes occurred in T1, T2, and T3, respectively. The corresponding numbers of episodes with treatment failure were 6 (5.3%), 14 (12.5%), and 30 (23.2%), respectively. As for the endpoint of catheter removal, the corresponding numbers were 4 (3.5%), 14 (12.5%), and 21 (18.8%), respectively. The NLR levels were higher in episodes with treatment failure than those with treatment success (*P* = 0.002). Similarly, the NLR levels were higher in episodes with catheter removal than those with catheter survival (*P* = 0.002, Figures 1A,B).

**Table 1:** Clinical characteristics of peritonitis episodes by NLR groups.

**Characteristic**	**Overall**	**NLR**		
		**T1 (<3.75)**	**T2 (3.75–6.53)**	**T3 (>6.53)**
Episode No.	337	113	112	112
Age, years	45 (21)	43 (20)	42 (23.5)	48 (18)
Male, *n* (%)	198 (58.8)	73 (64.6)	64 (57.1)	61 (54.5)
Hypertension, *n* (%)	309 (91.7)	103 (91.2)	101 (90.2)	105 (93.8)
Diabetes mellitus, *n* (%)	35 (10.4)	9 (8.0)	11 (9.8)	15 (13.4)
Coronary artery disease, *n* (%)	105 (31.2)	31 (27.4)	34 (30.4)	40 (35.7)
Duration on peritoneal dialysis, months	16 (25)	14 (24)	14 (22.5)	22.5 (26)
Residual urine volume, ml/24 h	300 (900)	500 (900)	300 (1,000)	200 (500)
Red blood cell, 10^12^/l	3.19 ± 0.72	3.24 ± 0.76	3.12 ± 0.65	3.21 ± 0.75
White blood cell, 10^9^/l	6.04 (3.78)	4.63 (2.24)	6.08 (3.77)	7.90 (4.97)
Platelet, 10^9^/l	204 (114)	196 (94)	204.5 (102)	219.5 (161.5)
Hemoglobin, g/l	94.58 ± 20.22	96.09 ± 20.59	94.18 ± 18.90	93.45 ± 21.18
Serum albumin, g/l	28.28 ± 6.89	29.65 ± 6.14	28.93 ± 6.82	26.18 ± 7.25
Serum creatinine, μmol/l	708 (326)	706 (293)	736.5 (293)	718.5 (409)
Serum uric acid, μmol/l	320 (112)	316 (99)	316.5 (119.5)	321 (131.5)
Serum cholesterol, mmol/l	3.74 (1.34)	3.81 (1.26)	3.74 (1.26)	3.71 (1.33)
Serum ferritin, μg/l	353 (416)	271 (400.1)	378.5 (409)	506.5 (425)
Serum potassium, mmol/l	3.78 (1.08)	3.87 (1.05)	3.65 (1.02)	3.61 (1.11)
Serum phosphorus, mmol/l	1.30 (0.57)	1.40 (0.48)	1.30 (0.52)	1.22 (0.65)
Dialysate white blood cell on day 3, 10^6^/l	153 (436)	161 (456)	149 (295.5)	162 (736.5)
Infection type, *n* (%)				
Gram-positive peritonitis	202 (59.9)	73 (64.6)	62 (55.4)	67 (59.8)
Gram-negative peritonitis	61 (18.1)	19 (16.8)	18 (16.1)	24 (21.4)
Culture-negative peritonitis	74 (22.0)	21 (18.6)	32 (28.6)	21 (18.8)
Treatment failure during episode, *n* (%)	46 (13.6)	6 (5.3)	14 (12.5)	26 (23.2)
Catheter removal during episode, *n* (%)	39 (11.6)	4 (3.5)	14 (12.5)	21 (18.8)
Death during episode, *n* (%)	7 (2.1)	2 (1.8)	0 (0)	5 (4.5)

*Episodes of peritoneal dialysis-associated peritonitis were grouped by tertiles (T1–3) of neutrophil-to-lymphocyte ratio*.

*NLR, neutrophil-to-lymphocyte ratio*.

### Association Between NLR and Treatment Failure

As shown in Table 2, we used GEE methods to assess the association between NLR and treatment failure events. After adjusting for age (OR, 1.01; 95% CI, 0.99–1.04; *P* = 0.205), PD duration (OR, 1.02; 95% CI, 1.00–1.03; *P* = 0.017), baseline WBCs (OR, 1.11; 95% CI, 1.02–1.21; *P* = 0.020), baseline serum albumin (OR, 0.95; 95% CI, 0.90–1.00; *P* = 0.037), baseline serum ferritin (OR, 1.04; 95% CI, 0.99–1.09; *P* = 0.081), dialysate WBCs on day 3 (OR, 1.03; 95% CI, 1.01–1.05; *P* = 0.002), and infection type [ORs of 1.80 (95% CI, 0.82–3.95; *P* = 0.142) and 1.74 (95% CI, 0.87–3.50; *P* = 0.142), respectively, for gram-negative and culture-negative peritonitis] (Supplemental Table 1), higher levels of NLR were independently associated with greater risks of treatment failure events with an adjusted OR value of 1.82 (95% CI, 1.05 to 3.15; *P* = 0.033) per natural log-transformed NLR. As a three-level categorical variable, in reference to the first tertile of NLR (T1), the risks of treatment failure events were higher, that the adjusted OR values were 2.37 (95% CI, 0.85 to 6.57; *P* = 0.098) for the second tertile (T2) and 3.41 (95% CI, 1.12–10.38; *P* = 0.030) for the third tertile (T3), respectively (*P* for trend = 0.026).

**Table 2:** The association between NLR and treatment failure in GEE models.

	**Odds ratio (95% confidence interval);** ***P*** **value**			
	**Crude**	**Model A**	**Model B**	**Model C**
Log-transformed NLR (per 1 unit increase)	2.37 (1.56–3.61); <0.001	2.15 (1.39–3.32);0.001	1.80 (1.06–3.08);0.031	1.82 (1.05–3.15);0.033
NLR tertiles				
T1	1.00 (reference)	1.00 (reference)	1.00 (reference)	1.00 (reference)
T2	2.55 (0.93–6.99);0.070	2.56 (0.93–7.00);0.068	2.49 (0.90–6.95);0.082	2.37 (0.85–6.57);0.098
T3	5.39 (2.13–13.67); <0.001	4.72 (1.80–12.38);0.002	3.41 (1.15–10.10);0.027	3.41 (1.12–10.38);0.030
*P* value for trend	<0.001	0.001	0.022	0.026

*Treatment failure was defined as catheter removal (including a temporary or permanent switch to hemodialysis) or death for any cause. Model A was adjusted for age and PD duration. Model B was adjusted for covariates in model A plus baseline WBCs, baseline serum albumin, baseline serum ferritin, and dialysate WBCs on day 3. Model C was adjusted for covariates in model B plus infection type. Infection type was expressed as a classified variable. Test for trend was done by treated NLR tertiles as an ordinal variable*.

*GEE, generalized estimation equation; NLR, neutrophil-to-lymphocyte ratio; PD, peritoneal dialysis; WBCs, white blood cell counts*.

**Figure 2:**
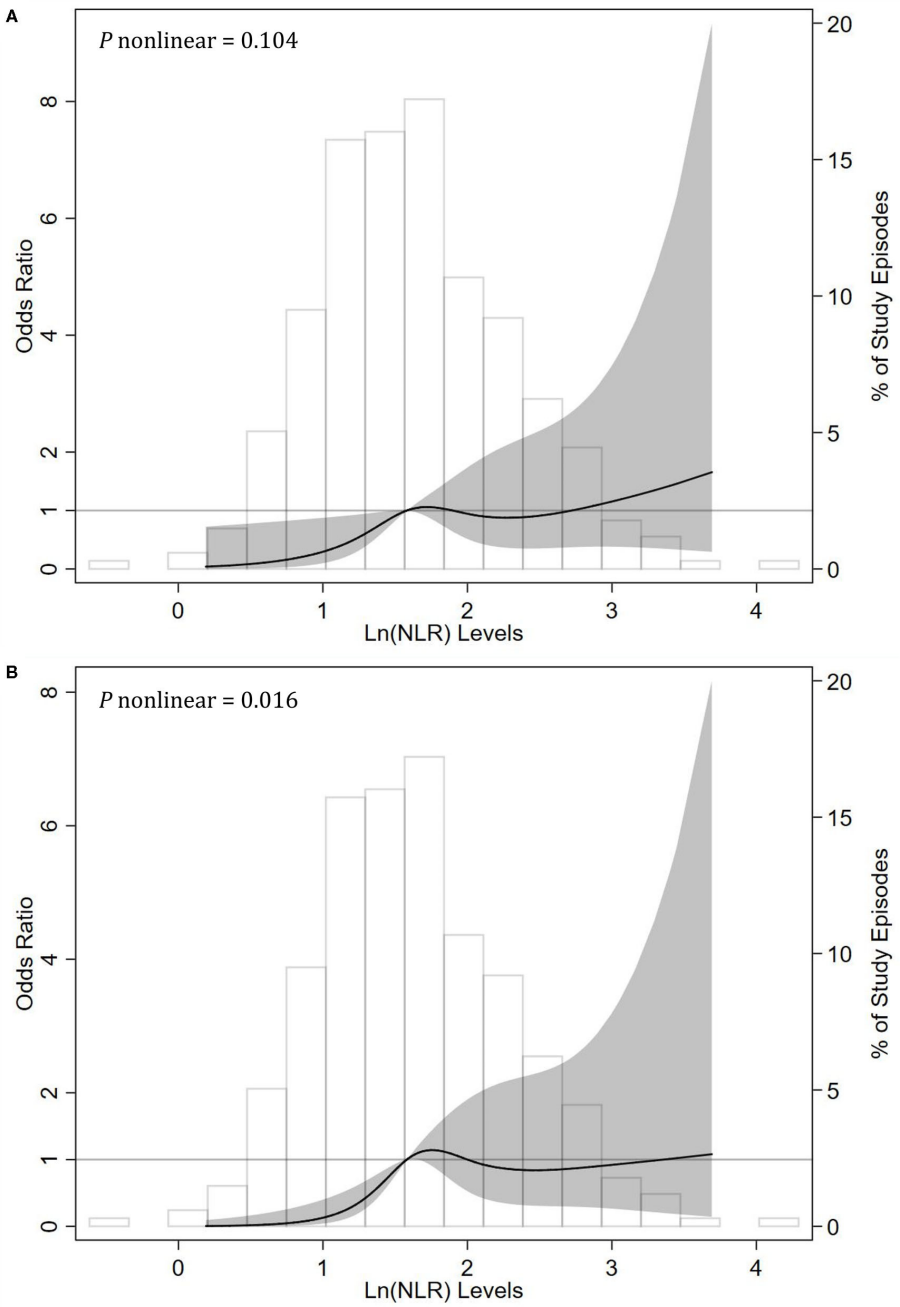
Associations between neutrophil-to-lymphocyte ratio (NLR) levels and risks of **(A)** treatment failure and **(B)** catheter removal events among episodes with peritonitis using restricted cubic spline (RCS) methods. RCSs were done with knots at the 25th (1.18), 50th (1.58), 75th (2.06), and 95th (2.87) percentiles of natural log-transformed NLR. The reference value (the gray lines) was set at the median. The solid black line indicates the trend of estimated odds ratios, the shaded area indicates the 95% confidence intervals, and the histogram represents the distribution of ln(NLR). The odds ratios of ln(NLR) in multivariable generalized estimation equation models were adjusted for age, duration on peritoneal dialysis, WBCs (white blood cell counts), serum albumin, serum ferritin, dialysate WBCs on day 3, and infection type (gram-positive, gram-negative, or culture-negative peritonitis).

To display the relationship between NLR levels and treatment failure in episodes of peritonitis, we modeled natural log-transformed NLR level as a continuous variable using RCS method with knots at the 25th, 50th, 75th, and 95th percentiles. After adjusting for other confounders, we found a linear association between the ln(NLR) levels and the risk of treatment failure events (*P* for non-linearity = 0.104). The risk of treatment failure events was greater with higher ln(NLR) levels (Figure 2A).

### Sensitivity Analyses

In sensitivity analyses, we recalculated the corresponding effect sizes, which excluded the effect of death and restricted the consequence to the catheter removal events. As shown in Table 3, after multivariable adjustment, NLR was a risk factor of catheter removal events with an OR value of 2.05 (95% CI, 1.14–3.69; *P* = 0.016) per natural log-transformed NLR. Compared with the first tertile (T1), the second (T2) and third tertiles (T3) of NLR substantially increased the risk of catheter removal events regardless of other confounders. The corresponding adjusted OR values were 3.96 (95% CI, 1.26–12.40; *P* = 0.018) and 5.85 (95% CI, 1.64–20.82; *P* = 0.006), respectively (*P* for trend = 0.004). However, in the RCS analysis, we observed a non-linear correlation of the ln(NLR) levels with the risk of treatment failure events (*P* for nonlinearity = 0.016, Figure 2B).

**Table 3:** The association between NLR and catheter removal in GEE models.

	**Odds ratio (95% confidence interval);** ***P*** **value**			
	**Crude**	**Model A**	**Model B**	**Model C**
Log-transformed NLR (per 1 unit increase)	2.16 (1.45–3.21); <0.001	1.96 (1.29–2.99);0.002	2.04 (1.15–3.60);0.014	2.05 (1.14–3.69);0.016
NLR tertiles				
T1	1.00 (reference)	1.00 (reference)	1.00 (reference)	1.00 (reference)
T2	3.82 (1.21–12.04);0.022	3.89 (1.26–12.05);0.018	4.18 (1.32–13.25);0.015	3.96 (1.26–12.40);0.018
T3	6.53 (2.19–19.50);0.001	5.82 (1.87–18.11);0.002	5.88 (1.69–20.49);0.005	5.85 (1.64–20.82);0.006
*P* value for trend	0.001	0.001	0.003	0.004

*Catheter removal was defined as a temporary or permanent switch to hemodialysis. Model A was adjusted for age and duration on PD. Model B was adjusted for covariates in model A plus baseline WBCs, baseline serum albumin, baseline serum ferritin, and dialysate WBCs on day 3. Model C was adjusted for covariates in model B plus infection type. Infection type was expressed as a classified variable. Test for trend was done by treated NLR tertiles as an ordinal variable*.

*GEE, generalized estimation equation; NLR, neutrophil-to-lymphocyte ratio; PD, peritoneal dialysis; WBCs, white blood cell counts*.

### Subgroup Analyses

In subgroup analyses (Table 4), the association between NLR levels and risk of treatment failure events was not significantly modified by age (>60 vs. ≤60 years; *P* for interaction = 0.843), gender (male vs. female; *P* for interaction = 0.253), PD duration (>12 vs. ≤12 months; *P* for interaction = 0.496), and serum albumin (>25 vs. ≤25 g/l; *P* for interaction = 0.961). However, this prognostic relevance was remarkably affected by the infection type (*P* for interaction = 0.047). Among episodes of gram-negative peritonitis, a higher NLR level substantially increased the incidence of treatment failure events (adjusted OR, 4.47; 95% CI, 1.34–14.97). Nevertheless, among episodes of nongram-negative peritonitis, the association between NLR levels and treatment failure events did not appear to be significant [adjusted OR values of 1.76 (95% CI, 0.81–3.80) and 3.84 (95% CI, 0.65–22.84), respectively, for gram-positive and culture-negative groups].

**Table 4:** Subgroup analyses of the association between NLR and treatment failure.

**Subgroup**	**Episode no./patient no**.	**Odds ratio (95% confidence interval)**		***P* value for interaction**
		**Crude**	**Adjusted**	
Total	337/202	2.37 (1.56–3.61)	1.82 (1.05–3.15)	
Age				0.843
>60 years	45/33	1.78 (0.67–4.73)	1.92 (0.36–10.23)	
≤60 years	292/169	2.50 (1.59–3.94)	1.85 (1.01–3.39)	
Gender				0.253
Male	198/120	2.28 (1.39–3.75)	1.88 (0.89–3.96)	
Female	139/82	2.63 (1.19–5.84)	2.02 (0.43–9.45)	
PD duration				0.496
>12 months	202/132	2.14 (1.27–3.59)	1.40 (0.67–2.91)	
≤12 months	135/99	2.47 (1.24–4.95)	3.92 (1.40–10.98)	
Serum albumin				0.961
>25 g/l	224/152	2.17 (1.32–3.57)	2.05 (0.99–4.24)	
≤25 g/l	113/83	2.50 (1.10–5.67)	3.34 (1.00–11.17)	
Infection type				0.047
Gram-positive peritonitis	202/133	1.70 (1.01–2.84)	1.76 (0.81–3.80)	
Gram-negative peritonitis	61/54	3.94 (1.82–8.57)	4.47 (1.34–14.97)	
Culture-negative peritonitis	74/62	3.45 (1.11–10.77)	3.84 (0.65–22.84)	

*GEE, generalized estimation equation; NLR, neutrophil-to-lymphocyte ratio; PD, peritoneal dialysis*.

## Discussion

The results of our study indicated that elevated NLR was a significant predictor of treatment failure events in episodes of single-bacteria PDAP, independent of other potential factors. Inflammatory status is highly common and related to adverse clinical outcomes including cardiovascular disease and all-cause mortality especially in patients who suffered ESRD or chronic dialysis (16–20). Inflammation under peritoneal dialysis was attributed to a few possible underlying reasons including the uremic microenvironment, infections, reduced clearance of pro-inflammatory cytokines, volume overload, oxidative stress, and some dialysis-associated factors (21–24). Inflammation can also interact with malnutrition and lead to the wastage and disorders in protein-energy nutritional status, resulting in the excessively high mortality in the dialysis population (22, 23). C-reactive protein (CRP), interleukin-6 (IL-6), and tumor necrosis factor-α (TNF-α) are among representative inflammatory markers; however, the detection of these markers in body fluids over time is an expensive task in current clinical practice. Finding an accessible and affordable inflammatory marker still seems a far way off from striking it.

To the best of our knowledge, this is the first study to demonstrate the association between NLR levels and adverse outcomes among patients with PDAP. NLR, an easily obtained parameter computed from complete blood count, is closely associated with inflammation and was originally treated as an oncologic prognostic indicator (25, 26). Afterwards, greater NLR levels have been linked to more severe inflammatory status and worse outcomes among patients with various disease conditions including cardiovascular disease, chronic obstructive pulmonary disease, liver cirrhosis, and even CKD (27–32). Recently, a few studies of dialysis patients suggested that NLR had moderate correlations with direct inflammatory markers such as CRP, IL-6, and TNF-α and that higher NLR levels were associated with greater mortality (12, 14, 15, 28). In this study, we displayed the relationship between NLR levels and treatment failure in episodes of PDAP using the RCS method. After adjusting for other confounders, we found a linear and positive association between the values of natural log-transformed (NLR) and the risk of treatment failure events (*P* for nonlinearity = 0.104). The underlying mechanisms of these findings have not been clearly elucidated, but the disclosure of the action pathways about inflammation may provide us with clues to explore what may provoke treatment failure events.

Another key finding from our study was that the relationship of NLR levels with risk of treatment failure events was significantly modified by infection type. Multiple studies have suggested the difference between gram-positive and gram-negative peritonitis in prognosis (33–35). A study from India suggested that catheter loss and hospital admission were significantly higher in gram-negative peritonitis than in gram-positive peritonitis (40.4 vs. 19.6%, *P* < 0.001; 62.9 vs. 41.2%, *P* = 0.004; respectively). However, mortality within 4 weeks was not statistically significant (21.3 vs. 9.8%) (36). The outcomes of single-organism peritonitis (gram-negative vs. gram-positive peritonitis) in PD in the Network 9 showed non-pseudomonal gram-negative peritonitis was associated with significantly more frequent catheter loss, hospitalization, and technique failure (37). Another study from Taiwan showed that, compared with gram-positive peritonitis, *Escherichia coli* peritonitis was associated with an increased risk of catheter removal even in younger female patients with PD (34). The overall incidence of treatment failure, catheter removal, and in-hospital mortality of *E. coli* peritonitis was 43, 38, and 9%, respectively. This means that, as an indicator of the inflammatory and nutritional status, NLR is more likely to play a predictive role in patients with gram-negative peritonitis. Meanwhile, this finding also underscores that more attention is needed to be paid in the management and prognostic assessment of gram-negative peritonitis. Additional research is warranted to control the persistent inflammatory state in gram-negative bacterial peritonitis and to improve the treatment outcomes of these patients.

Our research inevitably has several limitations: (1) due to the observational nature of the design, we can neither prove causality between NLR levels and risk of treatment failure events nor exclude the possibility of residual confounding; (2) the statistical analyses were based on a single measurement of laboratory parameters that may not reflect the association over time; (3) there are currently no established reference ranges for NLR values in the dialysis population. Our data showed a linear association between higher NLR and greater risk of treatment failure events in episodes of PDAP without clear thresholds indicating its rational ranges; (4) only the most common single-bacteria peritonitis were analyzed, and polymicrobial and special bacteria were not involved.

In general, our study suggested that increased NLR was independently associated with higher risks of treatment failure and catheter removal in episodes of PDAP. NLR, which is a convenient and inexpensive parameter, may be a novel marker of adverse outcomes among patients with peritonitis. Further research is needed to clarify the mechanism underlying the relationship of NLR with treatment failure and catheter removal and to identify effective anti-inflammatory treatments to prevent treatment failure events in patients with PDAP.

## Data Availability Statement

The raw data supporting the conclusions of this article will be made available by the authors, without undue reservation.

## Ethics Statement

This retrospective study was approved by the Ethics Committee of Xijing Hospital and performed in accordance with the Declaration of Helsinki and waived the need for informed consent because of the retrospective study design.

## Author Contributions

PH, JH, SS, CH, and LH designed the study, analyzed the data, and drafted the manuscript. PH and JH collected and entered data. SS, CH, and LH contributed to the data acquisition and interpretation. All authors read and approved the final manuscript.

## Conflict of Interest

The authors declare that the research was conducted in the absence of any commercial or financial relationships that could be construed as a potential conflict of interest.

## Publisher's Note

All claims expressed in this article are solely those of the authors and do not necessarily represent those of their affiliated organizations, or those of the publisher, the editors and the reviewers. Any product that may be evaluated in this article, or claim that may be made by its manufacturer, is not guaranteed or endorsed by the publisher.
