# Supplementary material for: Neutrophil-to-Lymphocyte Ratio and Treatment Failure in Peritoneal Dialysis-Associated Peritonitis
Source: Front Med (Lausanne). 2021 Jul 26;8:699502. doi: 10.3389/fmed.2021.699502 (PMC8350030; doi:10.3389/fmed.2021.699502)
Supplement: Supplementary file 1 [file Table_1.pdf]

Table S1. Univariable Analyses of Potential Predictors Related to Treatment Failure

Variable	Generalized Estimated Equation		
	**Odds Ratio**	**95% confidence interval**	***P* value**
Age, per yr increase	1.01	0.99-1.04	**0.205**
Gender (female)	0.81	0.44-1.51	0.511
Hypertension (yes)	0.94	0.35-2.55	0.909
Diabetes Mellitus (yes)	1.51	0.72-3.17	0.271
Coronary Artery Disease (yes)	1.08	0.58-2.03	0.810
Duration, per 1 mo increase	1.02	1.00-1.03	**0.017**
Residual Urine Volume, per 100 ml/24h increase	1.00	0.94-1.06	0.932
Red Blood Cell, per 10^{12}/liter increase	1.22	0.77-1.92	0.393
White Blood Cell, per 10^{9}/liter increase	1.11	1.02-1.21	**0.020**
Platelet, per 10^{11}/liter increase	1.07	0.90-1.28	0.460
Hemoglobin, per 1 g/liter increase	1.01	0.99-1.02	0.482
Serum Albumin, per 1 g/liter increase	0.95	0.90-1.00	**0.037**
Serum Creatinin, per 100 umol/liter increase	0.99	0.87-1.13	0.929
Uric Acid, per 100 umol/liter increase	1.07	0.80-1.44	0.629
Cholesterol, per 1 mmol/liter increase	0.84	0.60-1.17	0.298
Serum Ferritin, per 100 ug/liter increase	1.04	0.99-1.09	**0.081**
Potassium, per 1 mmol/liter increase	1.04	0.70-1.55	0.848
Phosphorus, per 1 mmol/liter increase	1.16	0.57-2.38	0.678
Dialysate White Blood Cell on Day 3, per 10^{8}/liter increase	1.03	1.01-1.05	**0.002**
Infection type			
Gram-positive peritonitis	1.00	referent	—
Gram-negative peritonitis	1.80	0.82 to 3.95	**0.142**
Culture negative peritonitis	1.74	0.87 to 3.50	**0.118**
